# Usability Methods and Attributes Reported in Usability Studies of Mobile Apps for Health Care Education: Scoping Review

**DOI:** 10.2196/38259

**Published:** 2022-06-29

**Authors:** Susanne Grødem Johnson, Thomas Potrebny, Lillebeth Larun, Donna Ciliska, Nina Rydland Olsen

**Affiliations:** 1 Faculty of Health and Function Western Norway University of Applied Sciences Bergen Norway; 2 Division of Health Services Norwegian Institute of Public Health Oslo Norway; 3 Faculty of Health Sciences McMaster University Hamilton, ON Canada

**Keywords:** user-computer interface, mobile apps, online learning, health education, students

## Abstract

**Background:**

Mobile devices can provide extendable learning environments in higher education and motivate students to engage in adaptive and collaborative learning. Developers must design mobile apps that are practical, effective, and easy to use, and usability testing is essential for understanding how mobile apps meet users’ needs. No previous reviews have investigated the usability of mobile apps developed for health care education.

**Objective:**

The aim of this scoping review is to identify usability methods and attributes in usability studies of mobile apps for health care education.

**Methods:**

A comprehensive search was carried out in 10 databases, reference lists, and gray literature. Studies were included if they dealt with health care students and usability of mobile apps for learning. Frequencies and percentages were used to present the nominal data, together with tables and graphical illustrations. Examples include a figure of the study selection process, an illustration of the frequency of inquiry usability evaluation and data collection methods, and an overview of the distribution of the identified usability attributes. We followed the Arksey and O’Malley framework for scoping reviews.

**Results:**

Our scoping review collated 88 articles involving 98 studies, mainly related to medical and nursing students. The studies were conducted from 22 countries and were published between 2008 and 2021. Field testing was the main usability experiment used, and the usability evaluation methods were either inquiry-based or based on user testing. Inquiry methods were predominantly used: 1-group design (46/98, 47%), control group design (12/98, 12%), randomized controlled trials (12/98, 12%), mixed methods (12/98, 12%), and qualitative methods (11/98, 11%). User testing methods applied were all think aloud (5/98, 5%). A total of 17 usability attributes were identified; of these, satisfaction, usefulness, ease of use, learning performance, and learnability were reported most frequently. The most frequently used data collection method was questionnaires (83/98, 85%), but only 19% (19/98) of studies used a psychometrically tested usability questionnaire. Other data collection methods included focus group interviews, knowledge and task performance testing, and user data collected from apps, interviews, written qualitative reflections, and observations. Most of the included studies used more than one data collection method.

**Conclusions:**

Experimental designs were the most commonly used methods for evaluating usability, and most studies used field testing. Questionnaires were frequently used for data collection, although few studies used psychometrically tested questionnaires. The usability attributes identified most often were satisfaction, usefulness, and ease of use. The results indicate that combining different usability evaluation methods, incorporating both subjective and objective usability measures, and specifying which usability attributes to test seem advantageous. The results can support the planning and conduct of future usability studies for the advancement of mobile learning apps in health care education.

**International Registered Report Identifier (IRRID):**

RR2-10.2196/19072

## Introduction

### Background

Mobile devices can provide extendable learning environments and motivate students to engage in adaptive and collaborative learning [1,2]. Mobile devices offer various functions, enable convenient access, and support the ability to share information with other learners and teachers [3]. Most students own a mobile phone, which makes mobile learning easily accessible [4]. However, there are some challenges associated with mobile devices in learning situations, such as small screen sizes, connectivity problems, and multiple distractions in the environment [5].

Developers of mobile learning apps need to consider usability to ensure that apps are practical, effective, and easy to use [1] and to ascertain that mobile apps meet users’ needs [6]. According to the International Organization for Standardization, usability is defined as “the extent to which a system, product or service can be used by specified users to achieve specified goals with effectiveness, efficiency, and satisfaction in a specified context of use” [7]. Better mobile learning usability will be achieved by focusing on user-centered design and attention to context, ensuring that the technology corresponds to the user’s requirements and putting the user at the center of the process [8,9]. In addition, it is necessary to be conscious of the interrelatedness between usability and pedagogical design [9].

A variety of usability evaluation methods exists to test the usability of mobile apps, and Weichbroth [10] categorized them into the following 4 categories: inquiry, user testing, inspection, and analytical modeling. Inquiry methods are designed to gather data from users through questionnaires (quantitative data) and interviews and focus groups (qualitative data). User testing methods include think-aloud protocols, question-asking protocols, performance measurements, log analysis, eye tracking, and remote testing. Inspection methods, in contrast, involve experts testing apps, heuristic evaluation, cognitive walk-through, perspective-based inspections, and guideline reviews. Analytical modeling methods include cognitive task analysis and task environment analysis [10]. Across these 4 usability evaluation methods, the most commonly used data collection methods are controlled observations and surveys, whereas eye tracking, think-aloud methods, and interviews are applied less often [10].

Usability evaluations are normally performed in a laboratory or in field testing. Previous reviews have reported that usability evaluation methods are mainly conducted in a laboratory, which means in a controlled environment [1,11]. By contrast, field testing is conducted in real-life settings. There are pros and cons to the 2 different approaches. Field testing allows data collection within a dynamic environment, whereas in a laboratory data collection and conditions are easier to control [1]. A variety of data collection methods are appropriate for usability studies; for instance, in laboratories, participants performing predefined tasks, such as using questionnaires and observations, are often applied [1]. In field testing, logging mechanisms and diaries have been applied to capture user interaction with mobile apps [1].

In all, 2 systematic reviews examined various psychometrically tested usability questionnaires as a means of enhancing the usability of apps. Sousa and Lopez [12] identified 15 such questionnaires and Sure [13] identified 13. In all, 5 of the questionnaires have proven to be applicable in usability studies in general: the System Usability Scale (SUS), Questionnaire for User Interaction Satisfaction, After-Scenario Questionnaire, Post-Study System Usability Questionnaire, and Computer System Usability Questionnaire [12]. The SUS questionnaire and After-Scenario Questionnaire are most widely applied [13]. The most frequently reported usability attributes of these 5 questionnaires are learnability, efficiency, and satisfaction [12].

Usability attributes are features that measure the quality of mobile apps [1]. The most commonly reported usability attributes are effectiveness, efficiency, and satisfaction [5], which are part of the usability definition [7]. In the review by Weichbroth [10], 75 different usability attributes were identified. Given the wide selection of usability attributes, choosing appropriate attributes depends on the nature of the technology and the research question in the usability study [14]. Kumar and Mohite [1] recommended that researchers present and explain which usability attributes are being tested when mobile apps are being developed.

Previous reviews have examined the usability of mobile apps in general [5,10,11,14,15]; however, only one systematic review has specifically explored the usability of mobile learning apps [1]. However, studies from health care education were not included. Similarly, usability has not been widely explored in medical education apps [16]. Thus, there is a need to develop a better understanding of how the usability of mobile learning apps developed for health care education has been evaluated and conceptualized in previous studies.

### Objectives

The aim of this scoping review has therefore been to identify usability methods and attributes in usability studies of mobile apps for health care education.

## Methods

### Framework

We have used the framework for scoping reviews developed by Arksey and O'Malley [17] and further developed by Levac et al [18] and Khalil et al [19]. We adopted the following five stages of this framework: (1) identifying the research question, (2) identifying relevant studies, (3) selecting studies, (4) charting the data, and (5) summarizing and reporting the results [17-19]. A detailed presentation of each step can be found in the published protocol for this scoping review [20]. We followed the PRISMA-ScR (Preferred Reporting Items for Systematic Reviews and Meta-Analyses extension for Scoping Reviews) checklist for reporting scoping reviews (Multimedia Appendix 1 [21]).

### Stage 1: Identifying the Research Question

The following two research questions have been formulated:

1. Which usability methods are used to evaluate the usability of mobile apps for health care education?
2. Which usability attributes are reported in the usability studies of mobile apps for health care education?

### Stage 2: Identifying Relevant Studies

A total of 10 electronic databases on technology, education, and health care from January 2008 to October 2021 and February 2022 were searched. These databases were as follows: Engineering Village, Scopus, ACM Digital Library, IEEE Xplore, Education Resource Information Center, PsycINFO, CINAHL, MEDLINE, EMBASE, and Web of Science. The search string was developed by the first author and a research librarian and then peer reviewed by another research librarian. The search terms used in the Web of Science, in addition to all relevant subject headings, included: ((student* or graduate* or undergraduate* or postgraduate*) NEAR/3 nurs*). This search string was repeated for other types of students and combined with the Boolean operator OR. The search string for all types of health care students was then combined with various search terms for mobile apps and mobile learning using the Boolean operator AND. Similar search strategies were used and adapted for all 10 databases as shown in Multimedia Appendix 2. In addition, a citation search in Google Scholar, screening reference lists of included studies, and searching for gray literature in OpenGrey were conducted.

### Stage 3: Selecting Studies

Two of the authors independently screened titles and abstracts using Rayyan web-based management software [22]. Studies deemed eligible by one of the authors were included for full-text screening and imported into the EndNote X9 (Clarivate) reference management system [23]. Eligibility for full-text screening was determined independently by two of the authors and disagreements were resolved by consensus-based discussions. Research articles with different designs were included, and there were no language restrictions. As mobile apps started appearing in 2008, this year was set as the starting point for the search. Eligibility criteria are presented in Table 1.

**Table 1:** Study eligibility.

	Inclusion criteria	Exclusion criteria
Population	Health care and allied health care students at the undergraduate and postgraduate levels	Health care professionals or students from education, engineering, or other nonhealth sciences
Concept	Studies of usability testing or methods of usability evaluation of mobile learning apps where the purpose relates to the development of the apps	Studies relating to learner management systems, e-learning platforms, open online courses, or distance education
Context	Typical educational setting (eg, classroom teaching, clinical placement, or simulation training), including both synchronous and asynchronous teaching	Noneducational settings not involving clinical placement or learning situations (eg, hospital or community settings)

### Stage 4: Charting the Data (Data Abstraction)

The extracted data included information about the study (eg, authors, year of publication, title, and country), population (eg, number of participants), concepts (usability methods, usability attributes, and usability phase), and context (educational setting). The final data extraction sheet can be found in Multimedia Appendix 3 [24-111]. One review author extracted the data from the included studies using Microsoft Excel software [21], which was checked by another researcher.

Descriptions of usability attributes have not been standardized, making categorization challenging. Therefore, a review author used deductive analysis to interpret the usability attributes reported in the included studies. This interpretation was based on a review of usability attributes as defined in previous literature. These definitions were assessed on the basis of the results of the included studies. This analysis was reviewed and discussed by another author. Disagreements were resolved through a consensus-based discussion.

### Stage 5: Summarizing and Reporting the Results

Frequencies and percentages were used to present nominal data, together with tables and graphical illustrations. For instance, a figure showing the study selection process, an illustration of the frequency of inquiry-based usability evaluation and data collection methods, and an overview of the distribution of identified usability attributes were provided.

## Results

### Eligible Studies

**Figure 1:**
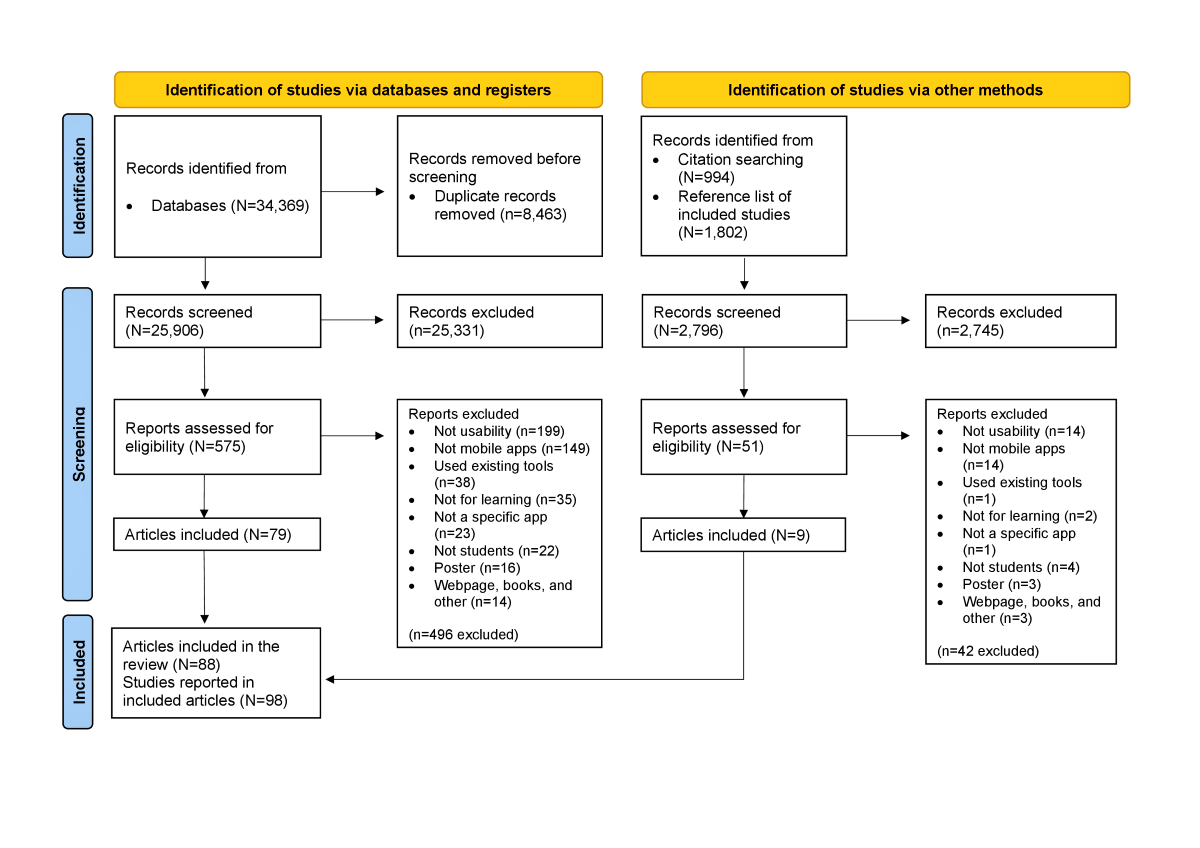
PRISMA (Preferred Reporting Items for Systematic Reviews and Meta-Analyses) flowchart of study selection process.

Database searches yielded 34,369 records, and 2796 records were identified using other methods. After removing duplicates, 28,702 records remained. A total of 626 reports were examined in full text. In all, 88 articles were included in the scoping review [24-111] (Figure 1). A total of 8 articles comprised results from several studies in the same article, presented as study A, study B, or study C in Multimedia Appendix 3. Therefore, a total of 98 studies were reported in the 88 articles included.

The included studies comprised a total sample population of 7790, with participant numbers ranging from 5 to 736 participants per study. Most of the studies included medical students (34/88, 39%) or nursing students (25/88, 28%). Other participants included students from the following disciplines: pharmacy (9/88, 10%), dentistry (5/88, 6%), physiotherapy (5/88, 6%), health sciences (3/88, 3%), and psychology (2/88, 2%). Further information is provided in Multimedia Appendix 3. There were 22 publishing countries, with most studies being from the United States (22/88, 25%), Spain (9/88, 10%), the United Kingdom (8/88, 9%), Canada (7/88, 8%), and Brazil (7/88, 8%), with an increasing number of publications from 2014. Table 2 provides an overview and characteristics of the included articles.

**Table 2:** Characteristics of included articles.

Study number	Study	Population (N)	Research design: data collection method	Usability attributes
1	Aebersold et al [24], 2018, United States	Nursing (N=69)	Mixed methods: questionnaire; task and knowledge performance^a^	Ease of use; learning performance; satisfaction; usefulness
2	Akl et al [25], 2008, United States	Resident (N=30)	Qualitative methods: focus groups; written qualitative reflections	Satisfaction
3	Al-Rawi et al [26], 2015, United States	Dentist (N=61)	Posttest 1-group design: questionnaire	Ease of use; frequency of use; satisfaction; usefulness
4	Albrecht et al [27], 2013, Germany	Medicine (N=6)	Posttest 1-group design: questionnaire^b^	Satisfaction
5	Alencar Neto et al [28], 2020, Brazil	Medicine (N=132)	Posttest 1-group design: questionnaire^b^	Ease of use; learnability; satisfaction; usefulness
6	Alepis and Virvou [29], 2010, Greece	Medicine (N=110)	Mixed methods: questionnaire; interviews	Ease of use; usefulness; user-friendliness
7	Ameri et al [30], 2020, Iran	Pharmacy (N=241)	Posttest 1-group design: questionnaire^b^	Context of use; efficiency; usefulness
8	Balajelini and Ghezeljeh [31], 2018, Iran	Nursing (N=41)	Posttest 1-group design: questionnaire	Ease of use; frequency of use; navigation; satisfaction; simplicity; usefulness
9	Barnes et al [32], 2015, United Kingdom	Medicine (N=42)	Randomized controlled trial: questionnaire; task and knowledge performance	Ease of use; effectiveness; learning performance; satisfaction
10	Busanello et al [33], 2015, Brazil	Dentist (N=62)	Pre-post test, nonrandomized control group design: questionnaire^b^	Learnability; learning performance; satisfaction
11	Cabero-Almenara and Roig-Vila [34], 2019, Spain	Medicine (N=50)	Pre-post test, 1-group design: questionnaire^b^	Learning performance; satisfaction
12	Choi et al [35], 2015, South Korea	Nursing (N=5)	Think-aloud methods: interviews; data from app	Context of use; ease of use; learnability; satisfaction; usefulness
13	Choi et al [36], 2018, South Korea	Nursing (N=75)	Pre-post test, nonrandomized control group design: questionnaire	Ease of use; learning performance; satisfaction; usefulness
14	Choo et al [37], 2019, Singapore	Psychology (N=8)	Mixed methods: questionnaire^b^; written qualitative reflections	Ease of use; learning performance; satisfaction; usefulness; user-friendliness
15	Chreiman et al [38], 2017, United States	Medicine (N=30)	Posttest 1-group design: questionnaire; data from app	Context of use; ease of use; frequency of use; usefulness
16	Colucci et al [39], 2015, United States	Medicine (N=115)	Posttest 1-group design: questionnaire	Effectiveness; efficiency; satisfaction; usefulness
17	Davids et al [40], 2014, South Africa	Residents (N=82)	Randomized controlled trial: questionnaire^b^; data from app	Effectiveness; efficiency; learnability; navigation; satisfaction; user-friendliness
18A	Demmans et al [41], 2018, Canada	Nursing (N=60)	Pre-post test, nonrandomized control group design: questionnaire; observations	Ease of use; effectiveness; learnability; learning performance; navigation; satisfaction
18B	Demmans et al [41], 2018, Canada	Nursing (N=85)	Pre-post test, nonrandomized control group design: questionnaire; observations	Ease of use; effectiveness; learnability; learning performance; navigation; satisfaction
19	Devraj et al [42], 2021, United States	Pharmacy (N=89)	Posttest 1-group design: questionnaire; data from app	Ease of use; errors; frequency of use; learning performance; navigation; operational usability; satisfaction; usefulness
20	Díaz-Fernández et al [43], 2016, Spain	Physiotherapy (N=110)	Posttest 1-group design: questionnaire	Comprehensibility; ease of use; usefulness
21	Docking et al [44], 2018, United Kingdom	Paramedic (N=24)	Think-aloud methods: focus groups	Context of use; learnability; satisfaction; usefulness
22	Dodson and Baker [45], 2020, United States	Nursing (N=23)	Qualitative methods: focus groups	Ease of use; operational usability; satisfaction; usefulness; user-friendliness
23	Duarte Filho et al [46], 2014, Brazil	Medicine (N=10)	Posttest nonrandomized control group design: questionnaire	Ease of use; efficiency; satisfaction; usefulness
24	Duggan et al [47], 2020, Canada	Medicine (N=80)	Posttest 1-group design: questionnaire; data from app	Ease of use; frequency of use; satisfaction; usefulness
25	Fernandez-Lao et al [48], 2016, Spain	Physiotherapy (N=49)	Randomized controlled trial: questionnaire^b^; task and knowledge performance	Learning performance; satisfaction
26	Fralick et al [49], 2017, Canada	Medicine (N=62)	Pre-post test, nonrandomized control group design: questionnaire	Ease of use; frequency of use; learning performance; usefulness
27	Ghafari et al [50], 2020, Iran	Nursing (N=8)	Posttest 1-group design: questionnaire	Ease of use; operational usability; satisfaction; usefulness
28	Goldberg et al [51], 2014, United States	Medicine (N=18)	Posttest 1-group design: questionnaire; task and knowledge performance	Ease of use; effectiveness
29	Gutiérrez-Puertas et al [52], 2021, Spain	Nursing (N=184)	Randomized controlled trial: questionnaire; task and knowledge performance	Learning performance; satisfaction
30	Herbert et al [53], 2021, United States	Nursing (N=33)	Randomized controlled trial: questionnaire; task and knowledge performance	Ease of use; learning performance; navigation; operational usability; usefulness
31	Hsu et al [54], 2019, Taiwan	Nursing (N=16)	Qualitative methods: interviews	Context of use; operational usability; satisfaction; usefulness
32	Huang et al [55], 2010, Taiwan	Not clear (N=28)	Posttest 1-group design: questionnaire	Ease of use; satisfaction, usefulness
33	Hughes and Kearney [56], 2017, United States	Occupational therapy (N=19)	Qualitative methods: focus groups	Efficiency; satisfaction
34	Ismail et al [57], 2018, Malaysia	Health science (N=124)	Pre-post test, 1-group design: questionnaire	Ease of use; learning performance; satisfaction; user-friendliness
35	Johnson et al [58], 2021, Norway	Occupational therapy, physiotherapy, and social education (N=15)	Qualitative methods: focus groups	Context of use; ease of use; operational usability
36A	Kang Suh [59], 2018, South Korea	Nursing (N=92)	Pre-post test, nonrandomized control group design: questionnaire; data from app	Effectiveness; frequency of use; learning performance; satisfaction
36B	Kang Suh [59], 2018, South Korea	Nursing (N=49)	Qualitative methods: focus groups	Effectiveness; frequency of use; learning performance; satisfaction
37	Keegan et al [60], 2016, United States	Nursing (N=116)	Posttest nonrandomized control group design: questionnaire; task and knowledge performance	Learning performance; satisfaction; usefulness
38	Kim-Berman et al [61], 2019, United States	Dentist (N=93)	Posttest 1-group design: questionnaire; task and knowledge performance	Context of use; ease of use; effectiveness; usefulness
39	Kojima et al [62], 2011, Japan	Physiotherapy and occupational therapy (N=41)	Pre-post test, 1-group design: questionnaire	Ease of use; learning performance; satisfaction; usefulness
40	Koulias et al [63], 2012, Australia	Medicine (N=171)	Posttest 1-group design: questionnaire	Ease of use; operational usability; satisfaction
41	Kow et al [64], 2016, Singapore	Medicine (N=221)	Pre-post test, 1-group design: questionnaire	Learning performance; satisfaction
42	Kurniawan and Witjaksono [65], 2018, Indonesia	Medicine (N=30)	Posttest 1-group design: questionnaire	Satisfaction; usefulness
43A	Lefroy et al [66], 2017, United Kingdom	Medicine (N=21)	Qualitative methods: focus groups; data from app	Context of use; frequency of use; satisfaction
43B	Lefroy et al [66], 2017, United Kingdom	Medicine (N=405)	Quantitative methods: data from app	Context of use; frequency of use; satisfaction
44	Li et al [67], 2019, Taiwan	Health care (N=70)	Pre-post test, nonrandomized control group design: questionnaire^b^	Ease of use; usefulness
45	Lin and Lin [68], 2016, Taiwan	Nursing (N=36)	Pre-post test, nonrandomized control group design: questionnaire	Cognitive load; ease of use; learnability; learning performance; usefulness
46	Lone et al [69], 2019, Ireland	Dentist (N=59)	Randomized controlled trial: questionnaire; task and knowledge performance	Ease of use; learnability; learning performance; operational usability; satisfaction
47A	Long et al [70], 2016, United States	Nursing (N=158)	Pre-post test, 1-group design: questionnaire; data from app	Ease of use; efficiency; learnability; learning performance; satisfaction
47B	Long et al [70], 2016, United States	Health science (N=159)	Randomized controlled trial: questionnaire; data from app	Ease of use; efficiency; learnability; learning performance; satisfaction
48	Longmuir [71], 2014, United States	Medicine (N=56)	Posttest 1-group design: questionnaire; data from app	Efficiency; learnability; operational usability; satisfaction
49	López et al [72], 2016, Spain	Medicine (N=67)	Posttest 1-group design: questionnaire^b^	Context of use; ease of use; errors; satisfaction; usefulness
50	Lozano-Lozano et al [73], 2020, Spain	Physiotherapy (N=110)	Randomized controlled trial: questionnaire; task and knowledge performance	Learning performance; satisfaction; usefulness
51	Lucas et al [74], 2019, Australia	Pharmacy (N=39)	Pre-post test, 1-group design: questionnaire; task and knowledge performance	Satisfaction; usefulness
52	Mathew et al [75], 2014, Canada	Medicine (N=5)	Think-aloud methods: questionnaire^b^; interviews; task and knowledge performance	Learnability; satisfaction
53	McClure [76], 2019, United States	Nursing (N=16)	Posttest 1-group design: questionnaire^b^	Learnability; satisfaction; usefulness
54	McDonald et al [77], 2018, Canada	Medicine (N=20)	Pre-post test, 1-group design: questionnaire; data from app	Effectiveness; satisfaction
55	McLean et al [78], 2014, Australia	Medicine (N=58)	Mixed methods: questionnaire; focus groups; interviews	Satisfaction
56	McMullan [79], 2018, United Kingdom	Health science (N=60)	Pre-post test, 1-group design: questionnaire	Learning performance; navigation; satisfaction; usefulness; user-friendliness
57	Mendez-Lopez et al [80], 2021, Spain	Psychology (N=67)	Pre-post test, 1-group design: questionnaire; task and knowledge performance	Cognitive load; ease of use; learning performance; satisfaction; usefulness
58	Meruvia-Pastor et al [81], 2016, Canada	Nursing (N=10)	Pre-post test, 1-group design: questionnaire; task and knowledge performance	Ease of use; learning performance; satisfaction; usefulness
59	Mettiäinen [82], 2015, Finland	Nursing (N=121)	Mixed methods: questionnaire; focus groups	Ease of use; usefulness
60	Milner et al [83], 2020, United States	Medicine and nursing (N=66)	Posttest 1-group design: questionnaire	Satisfaction; usefulness
61	Mladenovic et al [84], 2021, Serbia	Dentist (N=56)	Posttest 1-group design: questionnaire	Context of use; ease of use; satisfaction; usefulness
62	Morris and Maynard [85], 2010, United Kingdom	Physiotherapy and nursing (N=19)	Pre-post test, 1-group design: questionnaire	Context of use; ease of use; navigation; operational usability; usefulness
63A	Nabhani et al [86], 2020, United Kingdom	Pharmacy (N=56)	Posttest 1-group design: questionnaire	Ease of use; learnability; learning performance; satisfaction; usefulness
63B	Nabhani et al [86], 2020, United Kingdom	Pharmacy (N=152)	Posttest 1-group design: questionnaire	Ease of use; learnability; learning performance; satisfaction; usefulness
63C	Nabhani et al [86], 2020, United Kingdom	Pharmacy (N=33)	Posttest 1-group design: task and knowledge performance	Ease of use; learnability; learning performance; satisfaction; usefulness
64A	Noguera et al [87], 2013, Spain	Physiotherapy (N=84)	Posttest 1-group design: questionnaire	Learning performance; satisfaction; usefulness
64B	Noguera et al [87], 2013, Spain	Physiotherapy (N=76)	Randomized controlled trial: questionnaire	Learning performance; satisfaction; usefulness
65	O’Connell et al [88], 2016, Ireland	Medicine, nursing, and pharmacy (N=89)	Randomized controlled trial: questionnaire^b^	Ease of use; learning performance; operational usability; satisfaction; simplicity
66	Oliveira et al [89], 2019, Brazil	Medicine (N=110)	Randomized controlled trial: questionnaire; task and knowledge performance	Frequency of use; learning performance; satisfaction
67	Orjuela et al [90], 2015, Colombia	Medicine (N=22)	Posttest 1-group design: questionnaire	Ease of use; satisfaction
68	Page et al [91], 2016, United States	Medicine (N=356)	Mixed methods: questionnaire; interviews	Context of use; efficiency; satisfaction
69	Paradis et al [92], 2018, Canada	Medicine and nursing (N=108)	Posttest 1-group design: questionnaire^b^	Ease of use; satisfaction; usefulness
70	Pereira et al [93], 2017, Brazil	Medicine (N=20)	Posttest 1-group design: questionnaire^b^	Ease of use; learnability; satisfaction; usefulness
71	Pereira et al [94], 2019, Brazil	Nursing (N=60)	Posttest 1-group design: questionnaire	Ease of use; operational usability; satisfaction
72A	Pinto et al [95], 2008, Brazil	Biomedical informatics (N=5)	Qualitative methods: observations; task and knowledge performance	Efficiency; errors; learnability; learning performance; operational usability; satisfaction
72B	Pinto et al [95], 2008, Brazil	Medicine (N=not clear)	Posttest nonrandomized control group design: questionnaire	Efficiency; errors; learnability; learning performance; operational usability; satisfaction
73	Quattromani et al [96], 2018, United States	Nursing (N=181)	Randomized controlled trial: questionnaire^b^	Learnability; learning performance; satisfaction; usefulness
74	Robertson and Fowler [97], 2017, United States	Medicine (N=18)	Qualitative methods: focus groups	Satisfaction
75A	Romero et al [98], 2021, Germany	Medicine (N=22)	Think-aloud methods: questionnaire; interviews; task and knowledge performance	Effectiveness; efficiency; errors; navigation; satisfaction
75B	Romero et al [98], 2021, Germany	Medicine (N=22)	Posttest 1-group design: questionnaire^b^	Learnability; satisfaction
75C	Romero et al [98], 2021, Germany	Medicine (N=736)	Posttest 1-group design: questionnaire	Frequency of use; satisfaction
76	Salem et al [99], 2020, Australia	Pharmacy (N=33)	Posttest 1-group design: questionnaire	Operational usability; satisfaction; usefulness
77	San Martín-Rodríguezet al [100], 2020, Spain	Nursing (N=77)	Posttest 1-group design: questionnaire; task and knowledge performance	Learning performance; operational usability; satisfaction
78	Schnepp and Rogers [101], 2017, United States	Not clear (N=72)	Think-aloud methods: questionnaire^b^; interviews; task and knowledge performance	Learnability; satisfaction
79	Smith et al [102], 2016, United Kingdom	Medicine and nursing (N=74)	Mixed methods: questionnaire; focus groups	Navigation; operational usability; satisfaction; user-friendliness
80	Strandell-Laine et al [103], 2019, Finland	Nursing (N=52)	Mixed methods: questionnaire^b^; written qualitative responses	Learnability; operational usability; satisfaction
81	Strayer et al [104], 2010, United States	Medicine (N=122)	Mixed methods: questionnaire; focus groups	Context of use; learnability; learning performance; satisfaction; usefulness
82	Taylor et al [105], 2010, United Kingdom	A total of 8 different health care educations (N=79)	Qualitative methods: focus groups; written qualitative reflections	Context of use; learnability
83	Toh et al [106], 2014, Singapore	Pharmacy (N=31)	Posttest 1-group design: questionnaire	Ease of use; learnability; navigation; usefulness
84	Tsopra et al [107], 2020, France	Medicine (N=57)	Mixed methods: questionnaire; focus groups	Ease of use; operational usability; satisfaction; usefulness
85	Wu [108], 2014, Taiwan	Nursing (N=36)	Mixed methods: questionnaire; interviews	Cognitive load; effectiveness; satisfaction; usefulness
86	Wyatt et al [109], 2012, United States	Nursing (N=12)	Qualitative methods: focus groups	Ease of use; efficiency; errors; learnability; memorability; navigation; satisfaction
87	Yap [110], 2017, Singapore	Pharmacy (N=123)	Posttest 1-group design: questionnaire	Comprehensibility; learning performance; memorability; navigation; satisfaction; usefulness
88	Zhang et al [111], 2015, Singapore	Medicine (N=185)	Mixed methods: questionnaire; focus groups	Usefulness

^a^Performances measured, comparing paper and app results, quiz results, and exam results.

^b^Reported use of validated questionnaires.

### Usability Evaluation Methods

**Figure 2:**
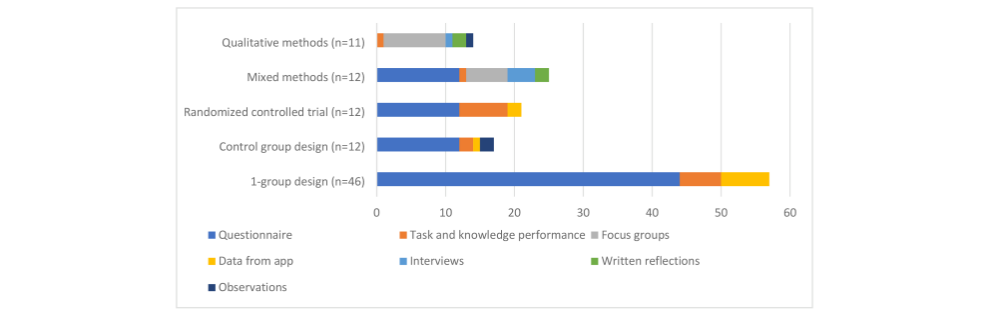
Inquiry usability evaluation methods and data collection methods.

The usability evaluation methods found were either inquiry-based or based on user testing. The following inquiry methods were used: 1-group design (46/98, 47%), control group design (12/98, 12%), randomized controlled trials (12/98, 12%), mixed methods (12/98, 12%), and qualitative methods (11/98, 11%). Several studies that applied inquiry-based methods used more than one data collection method, with questionnaires being used most often (80/98, 82%), followed by task and knowledge performance testing (17/98, 17%), focus groups (15/98, 15%), collection of user data from the app (10/98, 10%), interviews (5/98, 5%), written qualitative reflections (4/98, 4%), and observations (3/98, 3%). Additional information can be found in the data extraction sheet (Multimedia Appendix 3). Figure 2 illustrates the frequency of the inquiry-based usability evaluation methods and data collection methods.

The only user testing methods found were think-aloud methods (5/98, 5%), and 4 (80%) of these studies applied more than one data collection method. The data collection methods used included interviews (4/98, 4%), questionnaires (3/98, 3%), task and knowledge performance (3/98, 3%), focus groups (1/98, 1%), and collection of user data from the app (1/98, 1%).

A total of 19 studies used a psychometrically tested usability questionnaire, including the SUS, Technology Acceptance Model, Technology Satisfaction Questionnaire, and Technology Readiness Index. SUS [112] was used in most (9/98, 9%) of the studies.

Field testing was the most frequent type of usability experiment, accounting for 72% (71/98) of usability experiments. A total of 22 (22%) studies performed laboratory testing, and 5 (5%) studies did not indicate the type of experiment performed. Multimedia Appendix 3 provides an overview of the type of experiment conducted in each study. The usability testing of the mobile apps took place in a classroom setting (41/98, 42%), in clinical placement (29/98, 30%), during simulation training (14/98, 14%), other (7/98, 7%), or the setting was not specified (5/98, 5%).

### Usability Attributes

A total of 17 usability attributes have been identified among the included studies. The most frequently identified attributes were satisfaction, usefulness, ease of use, learning performance, and learnability. The least frequent were errors, cognitive load, comprehensibility, memorability, and simplicity. Table 3 provides an overview of the usability attributes identified in the included studies.

**Table 3:** Distribution of usability attributes (n=17) and affiliated reports (N=88).

Usability attribute	Distribution, n (%)	Reports (references)
Satisfaction	74 (84)	[24-28,31-37,39-42,44-48,50,52,54-57,59,60,62-66,69-81,83,84,86-104,107-110]
Usefulness	51 (58)	[24,26,28-31,35-39,42-47,49,50,53-55,60-62,65,67,68,72-74,76,79-87,92,93,96,99,104,106-108,110,111]
Ease of use	45 (51)	[24,26,28,29,31,32,35-38,41-43,45-47,49-51,53,55,57,58,61-63,67-70,72,80-82,84-86,88,90,92-94,106,107,109]
Learning performance	33 (38)	[24,32-34,36,37,41,42,48,49,52,53,57,59,60,62,64,68-70,73,79-81,86-89,95,96,100,104,110]
Learnability	23 (26)	[28,33,35,40,41,44,68-71,75,76,86,93,95,96,98,101,103-106,109]
Operational usability	19 (22)	[42,45,50,53,54,58,63,69,71,85,88,90,94,95,99-101,103,107]
Context of use	14 (16)	[30,35,38,44,54,58,61,66,72,84,85,91,104,105]
Navigation	12 (14)	[31,40-42,53,79,85,98,102,106,109,110]
Efficiency	11 (13)	[30,39,40,46,56,70,71,91,95,98,109]
Effectiveness	10 (11)	[32,39-41,51,59,61,77,98,108]
Frequency of use	10 (11)	[26,31,38,42,47,49,59,66,89,98]
User-friendliness	7 (8)	[29,37,40,45,57,79,102]
Errors	5 (6)	[42,72,95,98,109]
Cognitive load	3 (3)	[68,80,108]
Comprehensibility	2 (2)	[43,110]
Memorability	2 (2)	[109,110]
Simplicity	2 (2)	[31,88]

## Discussion

### Principal Findings

This scoping review sought to identify the usability methods and attributes reported in usability studies of mobile apps for health care education. A total of 88 articles, with a total of 98 studies reported in these 88 articles, were included in this review. Our findings indicate a steady increase in publications from 2014, with studies being published in 22 different countries. Field testing was used more frequently than laboratory testing. Furthermore, the usability evaluation methods applied were either inquiry-based or based on user testing. Most of the inquiry-based methods were experiments that used questionnaires as a data collection method, and all of the studies with user testing methods applied think-aloud methods. Satisfaction, usefulness, ease of use, learning performance, and learnability were the most frequently identified usability attributes.

### Comparison With Prior Work

#### Usability Evaluation Methods

The studies included in this scoping review mainly applied inquiry-based methods, primarily the collection of self-reported data through questionnaires. This is congruent with the results of Weichbroth [10], in which controlled observations and surveys were the most frequently applied methods. Asking users to respond to a usability questionnaire may provide relevant and valuable information. Among the 83 studies that used questionnaires in our review, only 19 (23%) used a psychometrically tested usability questionnaire; of these, the SUS questionnaire [112] was used most frequently. In line with the review on usability questionnaires [12], we recommend using a psychometrically tested usability questionnaire to support the advancement of usability science. As questionnaires address only certain usability attributes, mainly learnability, efficiency, and satisfaction [12], it would be helpful to also include additional methods, such as interviews or mixed methods, and to incorporate additional open-ended questions when using questionnaires.

Furthermore, the application of usability evaluation methods other than inquiry methods, such as user testing methods and inspection methods [10], could be beneficial and lead to more objective measures of app usability. Among other things, subjective data are collected via self-reported questionnaires, and objective data are collected based on task completion rates [40]. For example, in one of the included studies, the participants reported that the usability of the app was satisfactory by subjective measures, but the participants did not use the app [75]. Another study reported a lack of coherence between subjective and objective data; thus, these results indicate the importance of not relying solely on subjective measures of usability [40]. Therefore, it is suggested that various usability evaluation methods, including subjective and objective usability measures, are used in future usability studies.

Our review found that most of the included studies in health care education (71/98, 72%) performed field testing, whereas previous literature suggests that usability experiments in other fields are more often conducted in a laboratory [1,113]. For instance, Kumar and Mohite [1] found that 73% of the studies included in their review of mobile learning apps used laboratory testing. Mobile apps in health care education have been developed to support students’ learning, on-campus and during clinical placement, in various settings and on the move. Accordingly, it is especially important to test how the apps are perceived in specific environments [5]; hence, field testing is required. However, many usability issues can be discovered in a laboratory. Particularly in the early phases of app development, testing an app with several participants in a laboratory may make it more feasible to test and improve the app [8]. Usability testing in a laboratory can provide rapid feedback on usability issues, which can then be addressed before testing the app in a real-world environment. Therefore, it may be beneficial to conduct small-scale laboratory testing before field testing.

#### Usability Attributes

Previous systematic reviews of mobile apps in general identified satisfaction, efficiency, and effectiveness as the most common usability attributes [5,10]. In this review, efficiency and effectiveness were explored to a limited extent, whereas satisfaction, usefulness, and ease of use were the most frequently identified usability attributes. Our results coincide with those from a previous review on the usability of mobile learning apps [1], possibly because satisfaction, usefulness, and ease of use are usability attributes of particular importance when examining mobile learning apps.

Learning performance was assessed frequently in the included studies. For ensuring that apps are valuable in a given learning context, it is relevant to test additional usability attributes such as cognitive load [9]. However, few studies included in our review examined cognitive load [68,80,108]. Mobile apps are often used in an environment with multiple distractions, which may contribute to an increased cognitive load [5], affecting the learning performance. Testing both learning performance and app users’ cognitive load may improve the understanding of the app’s usability.

We found that several of the included studies did not use terminology from usability literature to describe which usability attributes they were testing. For instance, studies that tested satisfaction often used words such as “likes and dislikes” and “recommend use to others” and did not specify that they tested the usability attribute satisfaction. Specifying which usability attributes are investigated will be important when performing a usability study of mobile apps, as this will influence transparency and enable comparison between different studies. In addition, evaluating a wider range of usability attributes may enable researchers to expand their perspective regarding the app’s usability problems and ensure quicker improvement of the app. Defining and presenting different usability attributes in a reporting guideline can assist in deciding on and reporting relevant usability attributes. As such, a reporting guideline would be beneficial for researchers planning and conducting usability studies, a point that is also supported by the systematic review conducted by Kumar and Mohite [1].

### Future Directions

Combining different usability evaluation methods that incorporate both subjective and objective usability measures can add various and important perspectives when developing apps. In future studies, it would be advantageous to use psychometrically tested usability questionnaires to support the advancement of the usability science. In addition, developers of mobile apps should determine which usability attributes are relevant before conducting usability studies (eg, by registering a protocol). Incorporating these perspectives into the development of a reporting guideline would be beneficial to future usability studies.

### Strengths and Limitations

First, the search strategy was designed in collaboration with a research librarian and peer reviewed by another research librarian and included 10 databases and other sources. This broad search strategy resulted in a high number of references, which may be associated with a lower level of precision. To ensure the retrieval of all potentially pertinent articles, two of the authors independently screened titles and abstracts; studies deemed eligible by one of the authors were included for full-text screening.

Second, the full-text evaluation was challenging because the term *usability* has multiple meanings that do not always relate to usability testing. For instance, the term was used when testing students’ experience of a commercially developed app but not in connection with the app’s further development. In addition, many studies did not explicitly state that a mobile app was being investigated, which also created a challenge when deciding whether they satisfied the eligibility criteria. Nevertheless, reading the full-text articles independently by 2 reviewers and solving disagreements through consensus-based discussions ensured the inclusion of relevant articles.

### Conclusions

This scoping review was performed to provide an overview of the usability methods used and the attributes identified in usability studies of mobile apps in health care education. Experimental designs were commonly used to evaluate usability and most studies used field testing. Questionnaires were frequently used for data collection, although few studies used psychometrically tested questionnaires. Usability attributes identified most often were satisfaction, usefulness, and ease of use. The results indicate that combining different usability evaluation methods, incorporating both subjective and objective usability measures, and specifying which usability attributes to test seem advantageous. The results can support the planning and conduct of future usability studies of the advancement of learning apps in health care education.

## Appendix

Multimedia Appendix 1 PRISMA-ScR (Preferred Reporting Items for Systematic Reviews and Meta-Analyses extension for Scoping Reviews) checklist for reporting scoping reviews.

Multimedia Appendix 2 The search strategies for the 10 databases.

Multimedia Appendix 3 Data extraction sheet.

## Abbreviations

PRISMA-ScR: Preferred Reporting Items for Systematic Reviews and Meta-Analyses extension for Scoping Reviews
SUS: System Usability Scale
