# Supplementary material for: Usability Methods and Attributes Reported in Usability Studies of Mobile Apps for Health Care Education: Scoping Review
Source: JMIR Med Educ. 2022 Jun 29;8(2):e38259. doi: 10.2196/38259 (PMC9280458; doi:10.2196/38259)
Supplement: Multimedia Appendix 2 [file mededu_v8i2e38259_app2.docx]

**Multimedia Appendix 2.** The search strategies for the ten databases

Engineering Village, Scopus, ACM Digital Library, IEEE Xplore, Education Resource Information Center (ERIC), PsycINFO, CINAHL, Medline, Embase, and Web of Science.

| **Engineering Village** | | | | |
| --- | --- | --- | --- | --- |
| **Elsevier 2021 October 22** | | | | |
| **#** | **Query** | |  | |
| #1 | (((((( (((((student* NEAR/3 nurs*) OR (graduate* NEAR/3 nurs*) OR (postgraduate* NEAR/3 nurs*) OR (undergraduate* NEAR/3 nurs*))) AND (2008-2022 WN YR)) OR ((((student* NEAR/3 medical) OR (graduate* NEAR/3 medical) OR (postgraduate* NEAR/3 medical) OR (undergraduate* NEAR/3 medical))) AND (2008-2022 WN YR)) OR ((((student* NEAR/3 medicine) OR (graduate* NEAR/3 medicine) OR (postgraduate* NEAR/3 medicine) OR (undergraduate* NEAR/3 medicine))) AND (2008-2022 WN YR)) OR ((((student* NEAR/3 physiotherap*) OR (graduate* NEAR/3 physiotherap*) OR (postgraduate* NEAR/3 physiotherap*) OR (undergraduate* NEAR/3 physiotherap*))) AND (2008-2022 WN YR)) OR ((((student* NEAR/3 physical therap*) OR (graduate* NEAR/3 physical therap*) OR (postgraduate* NEAR/3 physical therap*) OR (undergraduate* NEAR/3 physical therap*))) AND (2008-2022 WN YR)) OR ((((student* NEAR/3 occupational therap*) OR (graduate* NEAR/3 occupational therap*) OR (postgraduate* NEAR/3 occupational therap*) OR (undergraduate* NEAR/3 occupational therap*))) AND (2008-2022 WN YR)) OR ((((student* NEAR/3 midwife*) OR (graduate* NEAR/3 midwife*) OR (postgraduate* NEAR/3 midwife*) OR (undergraduate* NEAR/3 midwife*))) AND (2008-2022 WN YR)) OR ((((student* NEAR/3 {social education*}) OR (graduate* NEAR/3 {social education*}) OR (postgraduate* NEAR/3 {social education*}) OR (undergraduate* NEAR/3 {social education*}))) AND (2008-2022 WN YR)) OR ((((student* NEAR/3 {social work*}) OR (graduate* NEAR/3 {social work*}) OR (postgraduate* NEAR/3 {social work*}) OR (undergraduate* NEAR/3 {social work*}))) AND (2008-2022 WN YR)) OR ((((student* NEAR/3 {social educator*}) OR (graduate* NEAR/3 {social educator*}) OR (postgraduate* NEAR/3 {social educator*}) OR (undergraduate* NEAR/3 {social educator*}))) AND (2008-2022 WN YR)) OR ((((student* NEAR/3 {medical technolog*}) OR (graduate* NEAR/3 {medical technolog*}) OR (postgraduate* NEAR/3 {medical technolog*}) OR (undergraduate* NEAR/3 {medical technolog*}))) AND (2008-2022 WN YR)) OR ((((student* NEAR/3 radiography) OR (graduate* NEAR/3 radiography) OR (postgraduate* NEAR/3 radiography) OR (undergraduate* NEAR/3 radiography))) AND (2008-2022 WN YR)) OR ((((student* NEAR/3 radiolog*) OR (graduate* NEAR/3 radiolog*) OR (postgraduate* NEAR/3 radiolog*) OR (undergraduate* NEAR/3 radiolog*))) AND (2008-2022 WN YR)) OR ((((student* NEAR/3 pharmac*) OR (graduate* NEAR/3 pharmac*) OR (postgraduate* NEAR/3 pharmac*) OR (undergraduate* NEAR/3 pharmac*))) AND (2008-2022 WN YR)) OR ((((student* NEAR/3 dentist*) OR (graduate* NEAR/3 dentist*) OR (postgraduate* NEAR/3 dentist*) OR (undergraduate* NEAR/3 dentist*))) AND (2008-2022 WN YR)) OR ((((student* NEAR/3 veterinar*) OR (graduate* NEAR/3 veterinar*) OR (postgraduate* NEAR/3 veterinar*) OR (undergraduate* NEAR/3 veterinar*))) AND (2008-2022 WN YR)) OR ((((student* NEAR/3 psycholog*) OR (graduate* NEAR/3 psycholog*) OR (postgraduate* NEAR/3 psycholog*) OR (undergraduate* NEAR/3 psycholog*))) AND (2008-2022 WN YR)) OR ((((student* NEAR/3 chiropract*) OR (graduate* NEAR/3 chiropract*) OR (postgraduate* NEAR/3 chiropract*) OR (undergraduate* NEAR/3 chiropract*))) AND (2008-2022 WN YR)) OR ((((student* NEAR/3 health*) OR (graduate* NEAR/3 health*) OR (postgraduate* NEAR/3 health*) OR (undergraduate* NEAR/3 health*))) AND (2008-2022 WN YR)) OR ((((student* NEAR/3 healthcare) OR (graduate* NEAR/3 healthcare) OR (postgraduate* NEAR/3 healthcare) OR (undergraduate* NEAR/3 healthcare))) AND (2008-2022 WN YR)))) AND ( ((((({mobile application} OR {mobile learning} OR mlearning OR m-learning OR {mobile phone} OR smartphone OR {personal digital assistant} OR smarphone* OR {smart phone*} OR {mobile phone*} OR {cell phone*} OR phone* OR telephone* OR {tablet* NEAR 3 computer*} OR {handheld computer*} OR {hand-held computer*} OR {handheld phone*} OR {hand-held phone*} OR {handheld device*} OR {hand-held device*} OR PDA* OR apps OR application* OR {learning app*} OR {application device*} OR {learning application*} OR {web 2.0}) AND (2008-2022 WN YR)) AND (((((nurs* OR medical OR medicine OR physioterap* OR physical therap* OR occupational therap* OR midwife* OR social education OR social work* OR social educator* OR medical technolog* OR radiography OR radiolog OR pharmac* OR dentist* OR veterinar* OR psycholog* OR chiropract* OR health* OR healthcare)) AND (2008-2022 WN YR)) AND (((student* OR graduate* OR postgraduate* OR undergraduate*)) AND (2008-2022 WN YR)))))))) AND ({ja} WN DT)))) | | |  |
| **Scopus** | | | | |
| **Elsevier 2021 October 22** | | | | |
| **#** | | **Query** |  | |
| #1 | | (((TITLE-ABS-KEY (((student* OR graduate* OR undergraduate* OR postgraduate*) W/3 nurs*))) OR (TITLE-ABS-KEY (((student* OR graduate* OR undergraduate* OR postgraduate*) W/3 medical))) OR (TITLE-ABS-KEY (((student* OR graduate* OR undergraduate* OR postgraduate*) W/3 medicine))) OR (TITLE-ABS-KEY (((student* OR graduate* OR undergraduate* OR postgraduate*) W/3 physiotherap*))) OR (TITLE-ABS-KEY (((student* OR graduate* OR undergraduate* OR postgraduate*) W/3 "physical therap*"))) OR (TITLE-ABS-KEY (((student* OR graduate* OR undergraduate* OR postgraduate*) W/3 "occupational therap*"))) OR (TITLE-ABS-KEY (((student* OR graduate* OR undergraduate* OR postgraduate*) W/3 midwife*))) OR (TITLE-ABS-KEY (((student* OR graduate* OR undergraduate* OR postgraduate*) W/3 "social work*"))) OR (TITLE-ABS-KEY (((student* OR graduate* OR undergraduate* OR postgraduate*) W/3 "social education"))) OR (TITLE-ABS-KEY (((student* OR graduate* OR undergraduate* OR postgraduate*) W/3 "social educator*"))) OR (TITLE-ABS-KEY (((student* OR graduate* OR undergraduate* OR postgraduate*) W/3   "medical technolog*"))) OR (TITLE-ABS-KEY (((student* OR graduate* OR undergraduate* OR postgraduate*) W/3 radiography))) OR(TITLE-ABS-KEY (((student* OR graduate* OR undergraduate* OR postgraduate*) W/3 radiolog*))) OR (TITLE-ABS-KEY (((student* OR graduate* OR undergraduate* OR postgraduate*) W/3 pharmac*))) OR (TITLE-ABS-KEY (((student* OR graduate* OR undergraduate* OR postgraduate*) W/3 dentist*))) OR (TITLE-ABS-KEY (((student* OR graduate* OR undergraduate* OR postgraduate*) W/3 veterinar*))) OR (TITLE-ABS-KEY (((student* OR graduate* OR undergraduate* OR postgraduate*) W/3 psycholog*))) OR (TITLE-ABS-KEY (((student* OR graduate* OR undergraduate* OR postgraduate*) W/3 health))) OR (TITLE-ABS-KEY (((student* OR graduate* OR undergraduate* OR postgraduate*) W/3 healthcare)))) AND ((TITLE-ABS-KEY ("mobile application*" OR "mobile learning" OR mlearning OR m-learning)) OR (TITLE-ABS-KEY (smartphone* OR "personal digital assistant" OR "smart phone*" OR "mobile phone*" OR "cell phone*" OR phone* OR telephone*)) OR (TITLE-ABS-KEY ((tablet W/3 computer*))) OR (TITLE-ABS-KEY ("handheld computer*" OR "hand-held computer*" OR "handheld phone*" OR "hand-held phone*" OR "handheld device*" OR "hand-held device*" OR pda*)) OR (TITLE-ABS-KEY (app OR apps OR application* OR "learning app*" OR "application device*" OR "learning application*" OR "web 2.0")))) AND NOT INDEX (medline) AND (LIMIT-TO (PUBYEAR, 2022) OR LIMIT-TO (PUBYEAR, 2021) OR LIMIT-TO (PUBYEAR, 2020) OR LIMIT-TO (PUBYEAR, 2019) OR LIMIT-TO (PUBYEAR, 2018) OR LIMIT-TO (PUBYEAR, 2017) OR LIMIT-TO (PUBYEAR, 2016) OR LIMIT-TO (PUBYEAR, 2015) OR LIMIT-TO (PUBYEAR, 2014) OR LIMIT-TO (PUBYEAR, 2013) OR LIMIT-TO (PUBYEAR, 2012) OR LIMIT-TO (PUBYEAR, 2011) OR LIMIT-TO (PUBYEAR, 2010) OR LIMIT-TO (PUBYEAR, 2009) OR LIMIT-TO (PUBYEAR, 2008)) |  | |

| **ACM Digital Library** | | |
| --- | --- | --- |
| **Elsevier 2022 March 01** | | |
| **#** | **Searches** |  |
| #1 | [[Abstract: student*] OR [Abstract: graduate*] OR [Abstract: undergraduate*] OR [Abstract: postgraduate*]] AND [[Abstract: medic*] OR [Abstract: nurs*] OR [Abstract: physiotherapy*] OR [Abstract: "physical therap*"] OR [Abstract: "occupational therap*"] OR [Abstract: midwife*] OR [Abstract: "social work*"] OR [Abstract: "social educa*"] OR [Abstract: "medical technology"] OR [Abstract: radiography] OR [Abstract: radiolog*] OR [Abstract: pharma*] OR [Abstract: dentis*] OR [Abstract: veterinary*] OR [Abstract: psychology*] OR [Abstract: chiropract*] OR [Abstract: health] OR [Abstract: healthcare]] AND [[Abstract: "mobile application*"] OR [Abstract: "mobile learning"] OR [Abstract: mlearning] OR [Abstract: "m -learning"] OR [Abstract: "mobile phone"] OR [Abstract: smartphone*] OR [Abstract: "personal digital assistant*"] OR [Abstract: smartphone*] OR [Abstract: "smart phone*"] OR [Abstract: "mobile phone*"] OR [Abstract: "cell phone*"] OR [Abstract: phone*] OR [Abstract: telephone*] OR [Abstract: tablet*] OR [Abstract: "handheld computer*"] OR [Abstract: "hand -held computer*"] OR [Abstract: "handheld phone*"] OR [Abstract: or] OR [Abstract: "hand -held phone*"] OR [Abstract: "handheld device*"] OR [Abstract: "hand -held device*"] OR [Abstract: pda*] OR [Abstract: app*] OR [Abstract: application*] OR [Abstract: "learning app*"] OR [Abstract: "application device*"] OR [Abstract: "learning application*"] OR [Abstract: "web 2.0"]] |  |
| #2 | Applied filters to #1: Proceedings and research article, Journals, and Year 2008-2022 |  |
| **IEEE Xplore** | | |
| **Elsevier 2022 February 22** | | |
| **#** | **Searches** |  |
| #1 | ((“student” OR "students" OR “graduate” OR "graduates" OR “postgraduate” OR "postgraduates" OR “undergraduate”)  NEAR/3 (“nurse” OR “medical” OR “medicine” OR “physiotherap*” OR “physical therapist” OR “occupational therapist” OR “midwife” OR “social worker” OR “social education” OR “social educator*” OR “medical technology” OR “radiography” OR “radiolog*” OR “pharmac*” OR “dentist” OR “veterinar*” OR “psycholog*” OR “chiropract*” OR “health” OR “healthcare”)) AND (("mobile learning" OR "mlearning" OR "m-learning" OR "smartphone" OR "smart phone" OR "mobile phone" OR "cell phone" OR "phone" OR "telephone" OR "tablet" OR "handheld computer" OR "hand-held computer" OR "handheld phone" OR "hand-held phone" OR "handheld device" OR "hand-held device" OR "PDA") OR ("app" OR "apps" OR "application" OR "applications" OR "learning app" OR "application device" OR "learning application" OR "web 2.0")) |  |
| #2 | Limit #1 to year 2008-2022 |  |

| **Education Resource Information Center (ERIC)** | | |
| --- | --- | --- |
| **EBSCOhost 2021 October 22** | | |
| **S** | **Searches** |  |
| S1 | ((student* or graduate* or postgraduate* or undergraduate*) N3 (nurs* or medical or medicine or physiotherap* or physical therap* or occupational therap* or midwife* or social work* or social education or social educator* or medical technolog* or radiography or radiolog* or pharmac* or dentist* or veterinar* or psycholog* or chiropract* or health or healthcare)) |  |
| S2 | DE "Nursing Students" OR DE "Medical Students" |  |
| S3 | S1 OR S2 |  |
| S4 | mobile learning or mlearning or m-learning |  |
| S5 | smartphone* or smart phone* or mobile phone* or cell phone* or phone* or telephone* |  |
| S6 | (tablet* n3 computer*) |  |
| S7 | handheld computer* or hand-held computer* or handheld phone* or hand-held phone* or handheld device* or hand-held device* or PDA* |  |
| S8 | app or apps or application* or learning app* or application device* or learning application* or web 2.0 |  |
| S9 | DE "Handheld Devices" |  |
| S10 | DE "Computer Oriented Programs" |  |
| S11 | S4 OR S5 OR S6 OR S7 OR S8 OR S9 OR S10 |  |
| S12 | S3 AND S11 |  |
| S13 | S3 AND S11, Limiters - Date Published: 20080101-20211231 |  |

| **PsycINFO** | | |
| --- | --- | --- |
| **PsycINFO 1806 to October Week 3 2021 via Ovid** | | |
| **#** | **Searches** |  |
| #1 | ((student* or graduate* or postgraduate* or undergraduate*) adj3 (nurs* or medical or medicine or physiotherap*  or physical therap* or occupational therap* or midwife* or social work* or social education or social educator* or medical technolog* or radiography or radiolog* or pharmac* or dentist* or veterinar* or psycholog* or chiropract* or health or healthcare)).ti,ab. |  |
| #2 | students/ or dental students/ or medical students/ |  |
| #3 | #1 or #2 |  |
| #4 | (mobile learning or mlearning or m-learning).ti,ab. |  |
| #5 | (smartphone* or smart phone* or mobile phone* or cell phone* or phone* or telephone*).ti,ab. |  |
| #6 | (tablet* adj3 computer*).ti,ab. |  |
| #7 | (handheld computer* or hand-held computer* or handheld phone* or hand-held phone* or handheld device* or hand-held device* or PDA*).ti,ab. |  |
| #8 | (app or apps or application* or learning app* or application device* or learning application* or web 2.0*).ti,ab. |  |
| #9 | mobile devices/ or cellular phones/ |  |
| #10 | #4 or #5 or #6 or #7 or #8 or #9 |  |
| #11 | #3 and #10 |  |
| #12 | limit #11 to yr="2008 -Current" |  |

| **Cumulative Index to Nursing and Allied Health Literature (Cinahl)** | | |
| --- | --- | --- |
| **EBSCOhost 2021 October 22** | |  |
| **S** | **Query** |  |
| S1 | ((student* or graduate* or postgraduate* or undergraduate*) N3 (nurs* or medical or medicine or physiotherap* or physical therap* or occupational therap* or midwife* or social work* or social education or social educator* or medical technolog* or radiography or radiolog* or pharmac* or dentist* or veterinar* or psycholog* or chiropract* or health or healthcare)) |  |
| S2 | (MH “Students, Health Occupations+”) |  |
| S3 | S1 OR S2 |  |
| S4 | (MH “Mobile Applications”) |  |
| S5 | mobile learning or mlearning or m-learning |  |
| S6 | (MH “Cellular Phone+”) OR (MH “Smartphone”) |  |
| S7 | (MH “Computers, Hand-Held”) OR (MH “Computers, Portable”) |  |
| S8 | smartphone* or smart phone* or mobile phone* or cell phone* or phone* or telephone* |  |
| S9 | (tablet* n3 computer*) |  |
| S10 | handheld computer* or hand-held computer* or handheld phone* or hand-held phone* or handheld device* or hand-held device* or PDA* |  |
| S11 | app or apps or application* or learning app* or application device* or learning application* or web 2.0 |  |
| S12 | S4 OR S5 OR S6 OR S7 OR S8 OR S9 OR S10 OR S11 |  |
| S13 | S3 AND S12 |  |
| S14 | S3 AND S12, Limiters - Published Date: 20080101-20211231 |  |

| **MEDLINE®** | | |  |  |
| --- | --- | --- | --- | --- |
| **MEDLINE 1946 to October 22, 2021 via Ovid** | | |  |  |
| **#** | | **Searches** |  | |
| #1 | | ((student* or graduate* or postgraduate* or undergraduate*) adj3 (nurs* or medical or medicine or physiotherapy* or physical therap* or occupational therap* or midwife* or social work* or social education or social educator* or medical technolog* or radiography or radiolog* or pharmac* or dentist* or veterinar* or psycholog* or chiropract* or health or healthcare)).ti,ab. |  | |
| #2 | | students, health occupations/ or students, dental/ or students, medical/ or students, nursing/ or students, pharmacy/ or students, public health/ |  | |
| #3 | | #1 or #2 |  | |
| #4 | | (mobile learning or mlearning or m-learning).ti,ab. |  | |
| #5 | | (smartphone* or smart phone* or mobile phone* or cell phone* or phone* or telephone*).ti,ab. |  | |
| #6 | | (tablet* adj3 computer*).ti.ab. |  | |
| #7 | | (handheld computer* or hand-held computer* or handheld phone* or hand-held phone* or handheld device* or hand-held device* or PDA*).ti,ab. |  | |
| #8 | | (app or apps or application* or learning app* or application device* or learning application* or web 2.0*).ti,ab. |  | |
| #9 | | Mobile Applications/ |  | |
| #10 | | Cell Phone/ |  | |
| #11 | | computers, handheld/ or smartphone/ |  | |
| #12 | | #4 or #5 or #6 or #7 or #8 or #9 or #10 or #11 |  | |
| #13 | | #3 and #12 |  | |
| #14 | | limit #13 to yr=»2008 -Current» |  | |
|  | |  |  | |
| **EMBASE** | | | |  |
| **Embase 1974 to 2021 October 22 via Ovid** | | | |  |
| **#** | **Searches** | |  |  |
| #1 | health student/ or medical student/ or public health student/ | |  |  |
| #2 | ((student* or graduate* or postgraduate* or undergraduate*) adj3 (nurs* or medical or medicine or physiotherap* or physical therap* or occupational therap* or midwife* or social work* or social education or social educator* or medical technolog* or radiography or radiolog* or pharmac* or dentist* or veterinar* or psycholog* or chiropract* or health or healthcare)).ti,ab. | |  |  |
| #3 | #1 or #2 | |  |  |
| #4 | mobile application/ | |  |  |
| #5 | (mobile learning or mlearning or m-learning).ti,ab. | |  |  |
| #6 | mobile phone/ or smartphone/ | |  |  |
| #7 | personal digital assistant/ | |  |  |
| #8 | (smartphone* or smart phone* or mobile phone* or cell phone* or phone* or telephone*).ti,ab. | |  |  |
| #9 | (tablet* adj3 computer*).ti,ab. | |  |  |
| #10 | (handheld computer* or hand-held computer* or handheld phone* or hand-held phone* or handheld device* or hand-held device* or PDA*).ti,ab. | |  |  |
| #11 | (app or apps or application* or learning app* or application device* or learning application* or web 2.0*).ti,ab. | |  |  |
| #12 | #4 or #5 or #6 or #7 or #8 or #9 or #10 or #11 | |  |  |
| #13 | #3 and #12 | |  |  |
| #14 | limit #13 to embase | |  |  |
| #15 | limit #14 to yr="2008 -Current" | |  |  |
|  |  | |  |  |
| **Web of Science** | | | |  |
| **All years: 1975-2021 October 22 via Clarivate Analytics** | | | |  |
| **#** | **Query** | |  |  |
| #1 | ((student* or graduate* or undergraduate* or postgraduate*) NEAR/3 nurs*) | |  |  |
| #2 | ((student* or graduate* or undergraduate* or postgraduate*) NEAR/3 (medical or medicine)) | |  |  |
| #3 | ((student* or graduate* or undergraduate* or postgraduate*) NEAR/3 physiotherap*) | |  |  |
| #4 | ((student* or graduate* or undergraduate* or postgraduate*) NEAR/3 "physical therap*") | |  |  |
| #5 | ((student* or graduate* or undergraduate* or postgraduate*) NEAR/3 "occupational therap*") | |  |  |
| #6 | ((student* or graduate* or undergraduate* or postgraduate*) NEAR/3 midwife*) | |  |  |
| #7 | ((student* or graduate* or undergraduate* or postgraduate*) NEAR/3 "social work*") | |  |  |
| #8 | ((student* or graduate* or undergraduate* or postgraduate*) NEAR/3 ("social education" or “social educator*”)) | |  |  |
| #9 | ((student* or graduate* or undergraduate* or postgraduate*) NEAR/3 "medical technolog*") | |  |  |
| #10 | ((student* or graduate* or undergraduate* or postgraduate*) NEAR/3 radiography) | |  |  |
| #11 | ((student* or graduate* or undergraduate* or postgraduate*) NEAR/3 radiolog*) | |  |  |
| #12 | ((student* or graduate* or undergraduate* or postgraduate*) NEAR/3 pharmac*) | |  |  |
| #13 | ((student* or graduate* or undergraduate* or postgraduate*) NEAR/3 dentist*) | |  |  |
| #14 | ((student* or graduate* or undergraduate* or postgraduate*) NEAR/3 veterinar*) | |  |  |
| #15 | ((student* or graduate* or undergraduate* or postgraduate*) NEAR/3 psycholog*) | |  |  |
| #16 | ((student* or graduate* or undergraduate* or postgraduate*) NEAR/3 health) | |  |  |
| #17 | ((student* or graduate* or undergraduate* or postgraduate*) NEAR/3 healthcare) | |  |  |
| #18 | #1 OR #2 OR #3 OR #4 OR #5 OR #6 OR #7 OR #8 OR #9 OR #10 OR #11 OR #12 OR #13 OR #14 OR #15 OR #16 OR #17 | |  |  |
| #19 | ("mobile application" OR "mobile learning" or mlearning or m-learning or "personal digital assistant" or smartphone* or "smart phone*" or "mobile phone*" or "cell phone*" or phone* or telephone* or (tablet* NEAR/3 computer*) or "handheld computer*" or "hand-held computer*" or "handheld phone*" or "hand-held phone*" or "handheld device*" or "hand-held device*" or PDA* or app or apps or application* or "learning app*" or "application device*" or "learning application*" or "web 2.0") | |  |  |
| #20 | #19 AND #18 | |  |  |
| #21 | #19 AND #18  Refined by: PUBLICATION YEARS: (2021 OR 2020 OR 2019 OR 2018 OR 2017 OR 2016 OR 2015 OR 2014 2013 OR 2012 OR 2011 OR 2010 OR 2009 OR 2008) | |  |  |
